# Comparing two mucin secretagogues for the treatment of dry eye disease: a prospective randomized crossover trial

**DOI:** 10.1038/s41598-024-63784-4

**Published:** 2024-06-10

**Authors:** Yeonwoo Jin, Kyoung Yul Seo, Sun Woong Kim

**Affiliations:** 1 Department of Ophthalmology, Yonsei University Wonju College of Medicine, 20 Ilsan-ro, Wonju, Gangwon-Do South Korea; 2 Department of Ophthalmology, Yonsei University College of Medicine, 50 Yonsei-ro, Seodaemun-gu, Seoul, South Korea

**Keywords:** Medical research, Eye manifestations

## Abstract

This study aimed to compare the clinical efficacy and investigate patients’ preferences for two mucin secretagogues in the treatment of dry eye disease (DED). Thirty patients with DED were randomly treated with either 3% diquafosol or 2% rebamipide ophthalmic solution for 4 weeks, followed by an additional 4-week treatment using the other eye drop after a 2-week washout period. Objective and subjective assessments, including the corneal and conjunctival staining score, tear breakup time (TBUT), Schirmer 1 test, tear osmolarity, tear matrix metalloproteinase-9 (MMP-9), lipid layer thickness (LLT) and ocular surface disease index (OSDI), were performed at baseline, 4 weeks, 6 weeks, and 10 weeks. Patient preferences were assessed based on four categories (comfort, efficacy, convenience, willingness to continue) using a questionnaire and the overall subjective satisfaction score for each drug was obtained at the end of the trial. In total, 28 eyes from 28 patients were included in the analysis. Both diquafosol and rebamipide significantly improved the OSDI (*p* = 0.033 and 0.034, respectively), TBUT (*p* < 0.001 and 0.026, respectively), and corneal (*p* < 0.001 and 0.001, respectively) and conjunctival (*p* = 0.017 and 0.042, respectively) staining after 4 weeks of treatment. An increase in Schirmer test scores was observed only after rebamipide treatment (*p* = 0.007). No significant changes were detected in tear osmolarity, MMP-9, and LLT following both treatments. The patients’ preference was slightly greater for diquafosol (46.4%) than rebamipide (36.7%), presumably due to rebamipide's bitter taste. The self-efficacy of both drugs and overall satisfaction scores were comparable. These findings indicate that two mucin secretagogues showed comparable effects in ameliorating symptoms and improving signs (TBUT, corneal and conjunctival staining) in patients with DED.

## Introduction

Dry eye disease (DED) is a multifactorial disease characterized by the loss of tear film homeostasis, which results in a variety of ocular symptoms^1,2^. In 2017, the Second Tear Film and Ocular Surface Society Dry Eye Workshop (TFOS-DEWS II) classified DED into three categories: aqueous deficient dry eye (ADDE), evaporative dry eye (EDE), and mixed form^1^. Tear hyperosmolarity and inflammation are the core mechanisms that lead to DED. In contrast, the Asia Dry Eye Society has placed more emphasis on tear film instability, which is measured by abnormal fluorescein tear breakup time (TBUT), as a diagnostic finding and has classified DED into ADDE, decreased wettability DE (DWDE), and increased evaporative DE (IEDE)^2^.

Based on the concept of tear film-oriented therapy, two mucin secretagogues, 3% diquafosol sodium, and 2% rebamipide ophthalmic suspensions have been widely used in Japan, as these drugs can replenish tear film layers and enhance tear film instability^3,4^. Both drugs have been reported to be effective for various kinds of DED including Sjogren’s syndrome, non-Sjogren ADDE, DWDE, and IEDE^5–9^. Diquafosol has also been used for decades in Korea, while rebamipide was recently introduced^10^. Although both drugs are known to increase the mucin levels of the ocular surface, their mechanisms of action are different. Diquafosol binds to P2Y_2_ receptors, increases the concentration of intracellular calcium, and results in the release of water and mucin from the conjunctival epithelial and goblet cells, respectively^11,12^. An increase in mucin and water levels is reportedly observed 15 min after drug instillation^13^. Furthermore, an increase in lipid layer thickness has also been reported after the installation of diquafosol^8,14^. Rebamipide was initially used to treat gastric ulcers, and the drug has been shown to increase mucin production and have anti-inflammatory effects in various tissues^15^. On the ocular surface, it increases the number of conjunctival goblet cells^16–18^, thereby increasing the secretory and membrane-associated mucins^19,20^. Previous in vitro studies have provided evidence regarding the anti-inflammatory effects of rebamipide and diquafosol. Kimura et al.^21^ reported that tumor necrosis factor alpha (TNF-α)-induced downregulation of Occludin (ZO-1), which is an indicator of inflammatory cytokine-caused damage to the tight junctions of corneal epithelial cells, was inhibited by rebamipide. Kim et al.^22^ reported that diquafosol attenuated the apoptosis of human corneal epithelial cells in hyperosmotic stress via downregulation of TNF-α and interleukin-6 (IL-6). However, the anti-inflammatory activities of rebamipide and diquafosol eye drops have not been demonstrated in clinical studies.

Only a limited number of studies have compared the clinical efficacies of diquafosol and rebamipide in the management of DED. The short-term effect of instillation showed that only diquafosol increased the MUC5AC levels in the tear fluid^13^, while long-term results demonstrated that both drugs increased the expression of MUC5AC in animal models^23^. A prospective randomized trial comparing these two drugs reported that both groups of drugs showed increased TBUT and symptom improvement^24^. Patients tend to prefer diquafosol over rebamipide, presumably due to the comfort associated with diquafosol use^24,25^. The major complaints associated with the use of rebamipide were a bitter taste and blurring. A newly developed rebamipide in Korea has been modified from its original Japanese composition. The Korean rebamipide ophthalmic solution is clear, not a suspension, and is known to reduce blurring after instillation^10^. Furthermore, the manufacturer claimed that the bitter taste was significantly reduced by the addition of mannitol and menthol.

Herein, this study compared the clinical effects of various parameters during dry eye treatment, such as the symptom score, dry eye-related signs, tear osmolarity, inflammation, and lipid layer thickness, along with patient preference for the two mucin secretagogues in a prospective crossover setting.

## Results

### Participants

Of the 30 participants enrolled, two were excluded from the analysis. The two excluded participants were first assigned diquafosol for use, and one dropped out due to discomfort from the first eye drop, and the other was excluded from the analysis because the use of systemic immunosuppressive drugs during the trial period was revealed. Finally, 28 eyes of 28 patients were included in the analysis. A total of 8 male and 20 female participants, with mean age of 61.3 ± 9.3 years, were included in the study. All patients were randomly treated with either diquafosol or rebamipide at the first visit and switched to the other medication at the third visit after a 2-week washout. The 13 participants who received diquafosol first had a mean age of 58.4 ± 8.7 years, and included 8 female participants (61.54%). The 15 participants who received rebamipide first had a mean age of 63.1 ± 9.4 years, and included 12 females (80.0%). There were no significant differences in age (*p* = 0.615 based on independent t test) and sex (*p* = 0.410 based on Fisher’s exact test). Baseline clinical parameters ​​before diquafosol and rebamipide use are shown in Table 1, with no significant differences revealed.

**Table 1:** Baseline clinical parameters before using each drug.

Baseline clinical parameters (n = 28, 28 eyes)	Diquafosol	Rebamipide	*p*-value*
OSDI	39.51 ± 22.28	36.31 ± 21.47	0.311
OSM	319.68 ± 21.97	329.46 ± 21.51	0.119
MMP9 (%)			
0	5 (17.9%)	5 (17.9%)	0.560
1	7 (25.0%)	8 (28.6%)	
2	15 (53.6%)	11 (39.3%)	
3	1 (3.6%)	4 (14.3%)	
Conjunctiva stain score	0.93 ± 1.33	1.11 ± 1.37	0.702
Cornea stain score	2.86 ± 1.90	2.86 ± 2.10	0.988
Schirmer test score	6.64 ± 2.36	6.75 ± 2.77	0.713
TBUT	3.04 ± 1.04	3.20 ± 1.19	0.617
LLT	69.29 ± 13.17	69.18 ± 18.53	0.982

*OSDI* ocular surface disease index, *OSM* tear osmolarity, *MMP9* Tear matrix metalloproteinase-9, *TBUT* tear breakup time, *LLT* lipid layer thickness.

*p*-value*: Independent t-test for OSM and TBUT, Mann–Whitney test for the other continuous variables, exact McNemar test for categorical variable.

### Changes in clinical parameters following diquafosol and rebamipide treatment

Significant improvements in the OSDI score were noted after the 4-week use of both diquafosol (*p* = 0.033, Table 2) and rebamipide (*p* = 0.034, Table 3). Both drugs showed a significant decrease in conjunctival (*p* = 0.017, Table 2 and *p* = 0.042, Table 3 for diquafosol and rebamipide, respectively) and corneal (*p* < 0.001, Table 2 and *p* = 0.001, Table 3 for diquafosol and rebamipide, respectively) staining scores. Significant prolongation of fluorescein TBUT was noted after both treatments (*p* < 0.001, Table 2 and *p* = 0.026, Table 3 for diquafosol and rebamipide, respectively). However, a significant increase in Schirmer’s test value was noted only for rebamipide (*p* = 0.007, Table 3). No significant differences were observed in the grades of tear MMP-9, tear osmolarity, and LLT (Tables 2, 3). There were no significant carry-over effects between the two treatment periods, and the observed treatment effects were not affected by the sequence of drug instillation or period effects (Table 4).

**Table 2:** Clinical parameters before and after treatment with diquafosol.

Clinical parameters (n = 28, 28 eyes)	Pre-treatment	Post-treatment	*p*-value*
OSDI	39.51 ± 22.28	32.54 ± 20.61	0.033
OSM	319.68 ± 21.97	319.14 ± 18.65	0.904
MMP9 (%)			
0	5 (17.9%)	2 (7.1%)	0.341
1	7 (25.0%)	10 (35.7%)	
2	15 (53.6%)	11 (39.3%)	
3	1 (3.6%)	5 (17.9%)	
Conjunctiva stain score	0.93 ± 1.33	0.46 ± 0.74	0.017
Cornea stain score	2.86 ± 1.90	1.32 ± 1.56	< 0.001
Schirmer test score	6.64 ± 2.36	8.07 ± 5.22	0.223
TBUT	3.04 ± 1.04	3.90 ± 1.02	< 0.001
LLT	69.3 ± 13.2	66.6 ± 12.8	0.361

*OSDI* ocular surface disease index, *OSM* tear osmolarity, *MMP9* Tear matrix metalloproteinase-9, *TBUT* tear breakup time, *LLT* lipid layer thickness.

*p*-value*: Paired t-test for OSM and TBUT, Wilcoxon signed rank test for the other continuous variables, exact McNemar test for categorical variable.

**Table 3:** Clinical parameters before and after treatment with rebamipide.

Clinical parameters (n = 28, 28 eyes)	Pre-treatment	Post-treatment	*p*-value*
OSDI	36.31 ± 21.47	28.13 ± 22.21	0.034
OSM	329.46 ± 21.51	320.93 ± 22.30	0.149
MMP9 (%)			
0	5 (17.9%)	4 (14.3%)	0.875
1	8 (28.6%)	9 (32.1%)	
2	11 (39.3%)	12 (42.9%)	
3	4 (14.3%)	3 (10.7%)	
Conjunctiva stain score	1.11 ± 1.37	0.61 ± 1.13	0.042
Cornea stain score	2.86 ± 2.10	1.54 ± 1.32	0.001
Schirmer test score	6.75 ± 2.77	9.18 ± 5.07	0.007
TBUT	3.20 ± 1.19	3.79 ± 1.16	0.026
LLT	69.2 ± 18.5	69.1 ± 15.9	0.923

*OSDI* ocular surface disease index, *OSM* tear osmolarity, *MMP9* Tear matrix metalloproteinase-9, *TBUT* tear breakup time, *LLT* lipid layer thickness.

*p*-value*: Paired t-test for OSM and TBUT, Wilcoxon signed rank test for the other continuous variables, exact McNemar test for categorical variable.

**Table 4:** Comparison of clinical measurements between the treatment groups and periods.

	Treatment period 1		Treatment period 2		P2	P3
	Visit1	Visit2	Visit3	Visit4		
OSDI						
Group A	38.81 ± 27.98	33.69 ± 26.13	41.46 ± 26.54	28.55 ± 25.22	0.272	0.766
Group B	31.83 ± 15.46	27.73 ± 20.13	40.11 ± 16.90	31.54 ± 15.22		
P1	0.786	0.717	0.964	0.413	0.573	
OSM						
Group A	321.38 ± 24.69	315.92 ± 18.29	330.08 ± 19.87	321.38 ± 19.30	0.547	0.302
Group B	328.93 ± 23.52	320.53 ± 25.28	318.20 ± 20.09	321.93 ± 19.13		
P1	0.415	0.590	0.129	0.940	0.526	
Conjunctiva stain score						
Group A	0.92 ± 1.66	0.31 ± 0.63	0.62 ± 1.04	0.15 ± 0.37	0.647	0.974
Group B	1.53 ± 1.51	1.07 ± 1.39	1.07 ± 1.03	0.73 ± 0.88		
P1	0.185	0.142	0.254	0.118	0.649	
Cornea stain score						
Group A	2.46 ± 1.56	1.00 ± 1.78	1.77 ± 1.48	1.00 ± 0.91	0.429	0.789
Group B	4.07 ± 2.15	2.07 ± 1.49	3.47 ± 2.59	2.00 ± 1.41		
P1	0.041	0.029	0.065	0.068	0.090	
Schirmer test score						
Group A	7.69 ± 3.30	8.46 ± 6.20	7.23 ± 3.09	9.15 ± 5.18	0.889	0.156
Group B	6.07 ± 2.22	9.47 ± 5.81	5.93 ± 2.31	7.93 ± 4.33		
P1	0.201	0.683	0.185	0.555	0.382	
TBUT						
Group A	2.95 ± 1.04	3.96 ± 1.15	3.45 ± 1.35	4.35 ± 1.36	0.932	0.703
Group B	2.93 ± 0.95	3.30 ± 0.77	3.17 ± 1.11	3.71 ± 0.92		
P1	0.956	0.083	0.548	0.167	0.084	
LLT						
Group A	68.23 ± 14.03	64.08 ± 10.32	71.92 ± 21.14	67.31 ± 17.48	0.845	0.921
Group B	65.93 ± 16.16	70.80 ± 13.71	69.73 ± 12.91	73.20 ± 15.61		
P1	0.294	0.363	0.751	0.294	0.057	

P1 between-group comparisons at each visit were performed using the independent t-test for OSM and TBUT and the Mann–Whitney test for the other continuous variables.

P1/P2 between-group comparison, P2 within-group comparison (time effect), P3 time × groups from generalized linear model.

Group A: treated with diquafosol during treatment period 1 then with rebamipide during treatment period 2. Group B: treated with rebamipide during treatment period 1 then with diquafosol during treatment period 2.

*OSDI* ocular surface disease index, *OSM* tear osmolarity, *MMP9* Tear matrix metalloproteinase-9, *TBUT* tear breakup time, *LLT* lipid layer thickness.

### Questionnaire regarding side effects

The questionnaire results are presented in Table 5. The discomfort score was significantly higher following the instillation of rebamipide (*p* = 0.001). The major complaint with rebamipide was its bitter taste. No significant differences were noted between the two drugs in terms of discharge, foreign body sensation, stinging sensation, and hyperemia in the eyes.

**Table 5:** Side effects of Diquafosol and Rebamipide according to the questionnaire.

Side effects (n = 28, 28 eyes)	Diquafosol	Rebamipide	*p*-value*
Discomfort score (0 ~ 5)	1.46 ± 1.45	2.82 ± 1.91	0.001
Discharge	10 (35.7%)	5 (17.9%)	0.113
Foreign body sensation	9 (32.1%)	6 (21.4%)	0.274
Stinging sensation	10 (35.7%)	5 (17.9%)	0.113
Bitter taste	2 (7.1%)	28 (100.0%)	< 0.001
Hyperemia	1 (3.6%)	2 (7.1%)	0.523

*p*-value* from Fisher’s exact test.

### Patient preference

**Figure 1:**
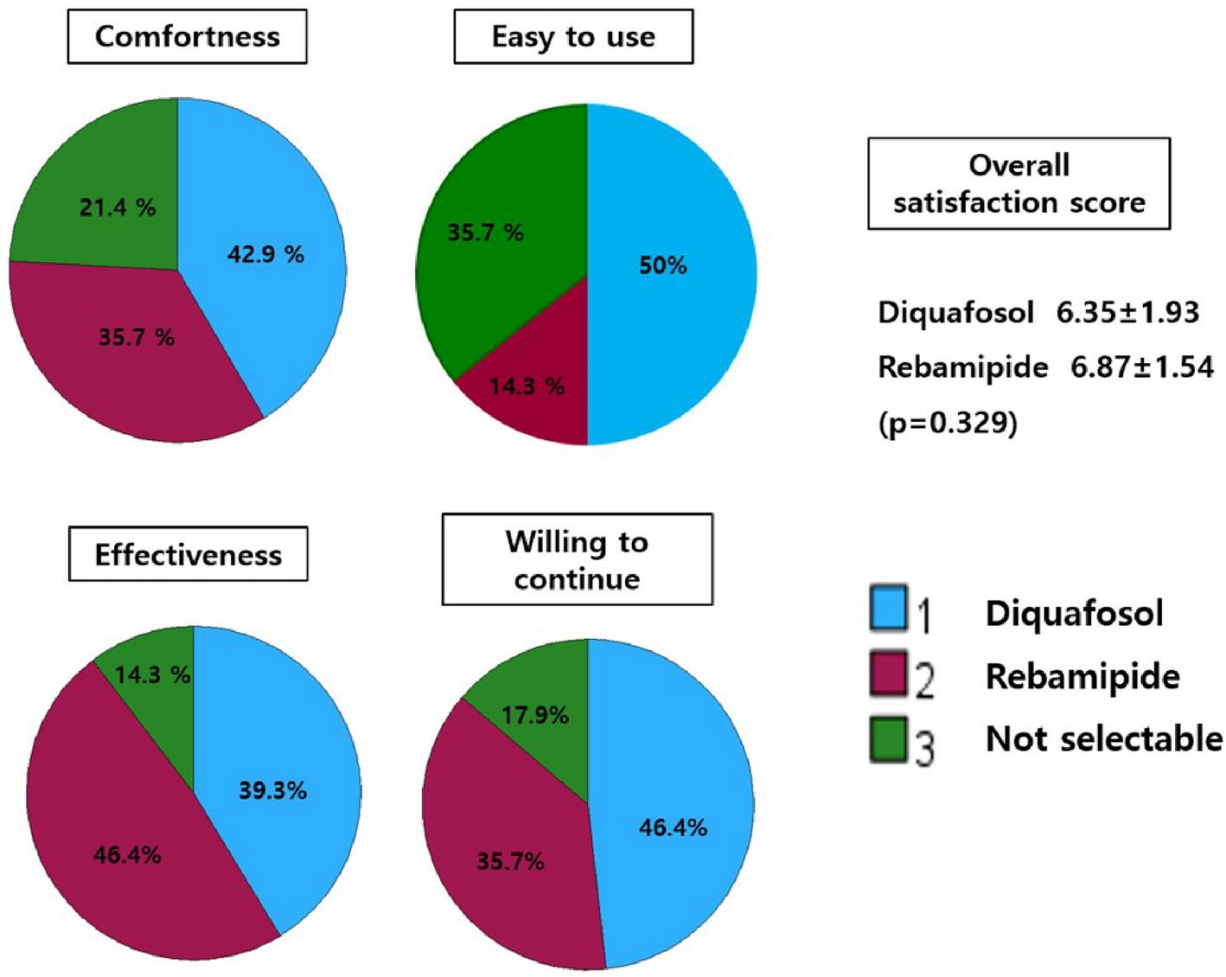
Patient preference for diquafosol and rebamipide.

Figure 1 shows patient preferences for diquafosol and rebamipide. There was no significant difference between the two drugs in terms of the overall satisfaction score (*p* = 0.329). A greater number of participants reported that diquafosol was easier to use and associated with more comfort in comparison to rebamipide. However, a greater number of participants reported that rebamipide was more effective than diquafosol, despite its bitter taste. When enquired about the choice of drug they would like to continue using, 46.4% (n = 13) chose diquafosol, 35.7% (n = 10) chose rebamipide, and 17.9% (n = 5) replied that they could not choose. The reasons for the indecision were that the effects of both drugs were either unsatisfactory or satisfactory. Two participants reported that they felt rebamipide was better in terms of efficacy but hesitated to continue using the drug due to its bitter taste. An additional analysis comparing pre-treatment parameters based on drug preference was conducted; however, no parameters associated with preference were identified (Table 6).

**Table 6:** Comparison of pre-treatment parameters according to drug preference.

Pre-treatment parameters	Diquafosol preferred (n = 13, 46.4%)	Rebamipide preferred (n = 10, 35.7%)	*p*-value
Proportion of using diquafosol first	7 (53.8%)	5 (50.0%)	0.660
Age	60.5 ± 7.2	63.10 ± 8.99	0.517
Sex (female)	11	6	0.183
OSM	323.31 ± 19.10	329.91 ± 21.96	0.563
OSDI	34.1 ± 19.7	33.71 ± 22.84	0.472
MMP9 (%)			
0	3 (23.1%)	2 (20.0%)	0.508
1	1 (7.7%)	2 (20.0%)	
2	8 (61.5%)	5 (50.0%)	
3	1 (7.7%)	1 (10.0%)	
Conjunctiva stain score	1.00 ± 1.63	0.80 ± 1.32	0.371
Cornea stain score	2.62 ± 1.98	3.00 ± 1.63	0.508
Schirmer test score	7.46 ± 3.26	7.30 ± 2.91	0.796
TBUT	3.08 ± 1.26	3.33 ± 1.35	0.648
LLT	64.69 ± 14.85	68.40 ± 13.07	0.752

*OSDI* ocular surface disease index, *OSM* tear osmolarity, *MMP9* Tear matrix metalloproteinase-9, *TBUT* tear breakup time, *LLT* lipid layer thickness.

*p*-value*: Independent t-test for age, OSM, and TBUT, Mann–Whitney test for the other continuous variables, Fisher’s exact test for categorical variable.

## Discussion

Artificial tears are the first-line therapy for DED, but in many cases, they are not sufficient as the sole medication^26,27^. The TFOS-DEWS II recommends a staged management algorithm implementing the various management and treatment options, including punctal occlusion, anti-inflammatory agents, oral macrolide or tetracycline, oral secretagogues, autologous serum, or other therapeutic modalities according to disease severity^28^. Although both diquafosol sodium and rebamipide ophthalmic solutions are representative mucin secretagogues, they differ in mechanisms of action and effects. Therefore, this study compared the clinical efficacy of two mucin-secretagogue eye drops and investigated patient preferences along with the possible factors associated with these preferences.

In this study, both drugs were found to be effective in recovering corneal and conjunctival staining, along with prolonging TBUT. Various studies have reported that diquafosol and rebamipide induce an increase in secretory- and membrane-associated mucins^13,19,29–31^. A major secretory mucin, MUC5AC, is known to reduce frictional stress through lubrication^32^ and clear allergens^33,34^. Membrane-associated mucins, primarily MUC1 and MUC16, provide non-adhesive barriers against pathogens^35–37^. Mucin also plays a role in prolonging TBUT and maintaining the viscosity of tear films^38,39^. Through this mechanism, the increase in mucin is thought to stabilize the tear film and aid in the healing of corneal and conjunctival epithelial damage.

When the magnitude of pre- and post-intervention changes were compared between the two drug groups, neither drug was found to have superior efficacy. A previous study that investigated the patient preference for diquafosol and rebamipide reported a higher preference for diquafosol (64.7%)^25^. The current study also showed a slightly higher preference for diquafosol (46.4%). This can be attributed to the bitter taste that frequently occurs after rebamipide instillation. Interestingly, a greater number of participants responded that the effect of rebamipide was better than that of diquafosol, despite the bitter taste or discomfort. This apparent conflict led to no significant difference in the overall satisfaction score between the two drugs. Unfortunately, this study failed to reveal any differential variables that might be useful for selecting one drug over another. A rapid increase in mucin and water content occurs after diquafosol instillation, whereas a gradual increase in secretory mucin content occurs due to an increase in the number of goblet cells after rebamipide instillation^11,13^. Rebamipide has been reported to be superior to diquafosol for relieving friction-related discomforts^40^. Thus, these drugs may complement each other.

The anti-inflammatory effects of both diquafosol and rebamipide have been demonstrated in vitro*.* However, there is a lack of evidence regarding the anti-inflammatory effects of the two drugs from human studies. MMP-9 is produced by the corneal epithelium, fibroblasts, and infiltrating leukocytes and is a well-known indicator of inflammation of the tear film^41–43^. Several studies have shown a reduction in MMP-9 levels after the use of diquafosol in type 2 diabetic dry eye patients^44^ or rebamipide in mouse models^45^. The tear MMP-9 level test is now available as an in-office test and is widely used to diagnose DED. This study investigated whether the two mucin secretagogues induce changes in tear MMP-9 levels using semi-quantitative analysis; however, no remarkable decrease in MMP-9 grades was observed after 4 weeks of treatment with either drug. This may have been due to the short treatment period or the limited anti-inflammatory effects of these drugs. It is possible that these drugs do not modulate MMP-9 production but exclusively regulate other inflammatory mediators. A relatively higher MMP-9 positivity rate in participants (average MMP9 positivity rate at visits 1, 2, 3, and 4 = 55.4%) should also be taken into consideration. Additionally, there were no significant changes in tear osmolarity after using either drug, suggesting that a 4-week treatment with a single eye drop may be insufficient to normalize tear osmolarity in these participants.

The current study had the strength of being the first to compare the clinical effects and preferences of two drugs in a crossover design where patients used both drugs. However, our study also had a few limitations that needed consideration. First, a significant increase in Schirmer’s test value was only observed after rebamipide use. Because diquafosol has been reported to increase tear volume in several studies^14,46,47^, our results were unexpected. However, since all clinical evaluations were performed at least 2 h after the instillation of either eye drop, the short-term water secretion effect of diquafosol may not have been reflected in our results. The lack of an increase in LLT in our study may be due to the interval between instillation and evaluation. Previous studies have indicated that an increase in LLT is maintained for up to 60 min in normal human eyes and in DED with MGD^8,14^. Second, a 4-week treatment may have been insufficient to induce some effects, such as reduced inflammation or osmolarity. Further studies are needed to determine the long-term effects of these drugs. Third, in this study, the excipients of the two studied drugs could not be matched, and this could be related to the findings. Specifically, the higher concentration of benzalkonium chloride (0.02% in rebamipide vs. 0.002% in diquafosol), which is known to be toxic to the ocular surface^48,49^, could have a potential association with the lower preference for rebamipide.

In conclusion, this study suggests that both diquafosol and rebamipide are effective in treating DED. Both drugs relieve patients’ symptoms by stabilizing the tear film and restoring the ocular surface epithelium. Therefore, both drugs can be widely used for the treatment of DED, depending on patient preferences in the clinical setting.

## Methods

### Study design and participants

This prospective, randomized, crossover clinical trial was conducted in Wonju, Republic of Korea, between December 2022 and June 2023. The trial was performed in accordance with the tenets of the Declaration of Helsinki. All procedures were approved by the Institutional Review Board of our hospital (Institutional Review Board of Wonju Severance Christian Hospital), and the study was registered with the Clinical Research Information Service (CRIS, KCT0008866) (12/10/2023). Written informed consent was obtained from all the participants before the commencement of the study.

Adult men and women aged ≥ 19 years, presenting with symptoms (e.g., foreign body sensation, dryness, photophobia, eye pain, blurred vision) suggestive of DED for more than 3 months and meeting all of the following criteria were included in this trial: (1) Schirmer I test ≤ 10 mm/5 min; (2) Fluorescein TBUT ≤ 10 s; and (3) corneal and conjunctival staining score by National Eye Institute (NEI) grading ≥ 1 point. Patients who met any of the following criteria were excluded from the study: (1) history of refractive surgery within 1 year; (2) history of any ocular surgery within 3 months; (3) history of punctal plug insertion or punctal occlusion within 3 months; (4) use of systemic steroids, immunosuppressive drugs, steroid eye drops, or cyclosporine eye drops; and (5) intraocular pressure (IOP) ≥ 22 mmHg in one or both eyes or presence of untreated glaucoma.

The sample size was estimated for non-inferiority tests based on 2 × 2 cross-over design using a sample size calculator provided by the Center for Clinical Research and Biostatics (CCRB, https://www2.ccrb.cuhk.edu.hk/stat/mean/tsmc_sup.htm#2). When the non-inferiority margin of TBUT is defined as 0.5, the true difference between the means is assumed to be 0, with population variance of 1.0 and a given the significance level of α = 0.05; here, the sample size to achieve 80% power is n = 13 for each group. As loss to follow-up can occur, considering a drop-out rate of 15%, 30 total participants were recruited for this study. Patients were enrolled if they met the criteria through a screening test at the first visit (visit 1) and were randomly assigned to two groups that were started with one of two mucin secretagogues, as follows: 3% diquafosol sodium eye drops (SCD pharm, Seoul, Korea) in Group A or 2% rebamipide eye drops (Kukje pharma, Sungnam-si, Gyeonggi-do, Korea) in Group B. Before enrollment, the group assignment sequence, established by flipping a coin, was as follows: B-A-B-B-A-A-B-B-A-B-A-A-A-B-A-B-A-B-B-B-A-A-B-A-A-B-B-A-B-A. Participants were assigned to each group in line with their visit order. One of the investigational products was issued and retrieved after 4 weeks by the pharmacy, and the researchers and participants were blinded to drug information during clinical trials. If patients used artificial tears during the screening phase, the protocol was started after a 2-week washout period and the use of artificial tears was discontinued during the entire study period. The study participants were instructed to use the eye drops four times a day, and after 4 weeks of use, a second visit (visit 2) was scheduled to evaluate the effectiveness of the first drug and conduct a questionnaire session regarding the drug side effects with an overall discomfort score. After a 2-week washout period, the patient visited the hospital for the third time (visit 3), and a new baseline test was performed. The participants were instructed to use the other drug four times a day for an additional 4 weeks. At the last visit (visit 4), the effect of the second drug was evaluated, and a questionnaire session on drug side effects was conducted. At the last visit, a survey was conducted asking patients to choose their preference between the two medications in terms of comfort, convenience, self-efficacy, and future use, with an overall satisfaction score ranging between 1 and 10.

### Clinical testing

Participants were instructed not to use eye drops for at least 2 h before each visit. At every visit (visits 1, 2, 3, and 4), the participants underwent standard ophthalmic examination, including measurement of IOP, slit lamp examination, ocular surface disease index (OSDI) questionnaire (OSDI; Allergan, Irvine, CA, USA), and subsequently clinical investigations. To assess dry eye symptoms, a 12-item OSDI questionnaire, which consists of three subscales (ocular symptoms, vision-related functions, and environmental triggers) during a 1-week recall period was completed^50^. To evaluate tear film stability, a sterile fluorescein paper strip (Haag-Streit, Bern, Switzerland) wetted with normal saline was applied to the inferior fornix. Two minutes after fluorescein application, the interval between the last complete blink and the first appearance of dark spots in the tear film was recorded using a slit-lamp microscope with a cobalt-blue filter as the TBUT^51^. The mean value of 3 consecutive measurements was documented. Corneal fluorescein staining was evaluated using the NEI scale, which relies on a chart that divides the cornea into five sections and assigns a value from 0 (absent) to 3 (severe) to each section, based on the amount, size, and confluence of punctate keratitis, to obtain a maximum score of 15 points. Meanwhile conjunctival staining was evaluated using lissamine green paper strips (Contacare Ophthalmics & Diagnostics, Padra, India) and graded using the NEI scale from 0 (absent) to 3 (severe) for each of the six areas on each conjunctiva, for a maximum score of 18 points^52^. The Schirmer I test was performed to measure tear volume. Tear matrix metalloproteinase-9 (MMP-9) was evaluated using a point-of-care MMP-9 immunoassay (InflammaDry, Quidel, CA, USA) following the manufacture’s instruction. Briefly, the sampling fleece was dabbed 8–10 times in multiple locations until it was saturated. The test was assembled by placing the fleece of the sample collector into the sample transfer window of the test cassette body. The absorbent tip was immersed into the buffer vial for 20 s and laid flat on a horizontal surface for 10 min. The test was read thereafter under brightly lit conditions and reread after 10 min for negative results as recommended by the manufacturer. For semi-quantitative analysis of tear MMP-9 level, 5-scale grades ranging from 0 to 4 were used^53^. Tear osmolarity was measured with the I-PEN tear osmolarity system (I-MED Pharma, Dollard-des-Ormeaux, Quebec, Canada). The measurement was performed by the same investigator in the same examination room with a controlled temperature of 23.5–26.0 °C and humidity of 35–40%. Following gentle eyelid closure for 30–60 s, the tip of the single-use sensor (SUS) was placed at a 30°–45° angle directly onto the palpebral conjunctiva on the inside of the retracted lower eyelid, with the gold node from the SUS in good contact with the palpebral conjunctiva. After a few seconds in this position, the handheld osmolarity system generates an audible beep and displays the osmolarity reading in milliosmole per liter on its LCD screen^54^. A lipid layer thickness (LLT) test was performed using IDRA (SBM Sistemi, Torino, Italy). The device projects white light over the cornea, and the light reflected from the tear film can be observed as a white fan-shaped area that covers the lower third of cornea. The automatic interferometry test of IDRA detects the interference of colors from the lipid layer on the tear film. Further, it determines the average, maximum, and minimum LLT using the international grade scale of Dr. Guillon with the thicknesses related to each grade of the lipid layer pattern^55,56^. Depending on the patterns, the grades were converted to nanometers and could be classified between 15 and 100 nm.

### Statistical analysis

The primary efficacy outcome was TBUT, and the secondary outcomes were OSDI, Schirmer test, corneal and conjunctival staining score and LLT. Moreover, preference was the main outcome of this study. Data from randomly selected eyes from each participant were used for statistical analyses. The Kolmogorov–Smirnov test was performed to determine normality and to compare the parameters before and after use of the two drugs, the paired t-test was performed for OSM and TBUT and Wilcoxon signed rank test was performed for other continuous variables. Either the Fisher’s exact test or the exact McNemar test was used for categorical variables. Comparisons of the treatment effects based on the group and treatment periods were conducted using the generalized linear model. Baseline characteristics before the instillation of the two eye drops at visits 1 and 3 were also compared using an independent t-test or the Mann–Whitney test to investigate possible differences over time. Statistical analyses were performed using SPSS version 26.0 (SPSS Inc., Chicago, IL, USA). The level of significance was set at a *p*-value < 0.05.

## Data Availability

The datasets used and/or analysed during the current study available from the corresponding author on reasonable request.
